# A Nomogram Predicts Individual Prognosis in Patients With Newly Diagnosed Glioblastoma by Integrating the Extent of Resection of Non-Enhancing Tumors

**DOI:** 10.3389/fonc.2020.598965

**Published:** 2020-12-02

**Authors:** Zhe Zhang, Zeping Jin, Dayuan Liu, Yang Zhang, Chunzhao Li, Yazhou Miao, Xiaohan Chi, Jie Feng, Yaming Wang, Shuyu Hao, Nan Ji

**Affiliations:** ^1^ Department of Neurosurgery, Beijing Tiantan Hospital, Capital Medical University, Beijing, China; ^2^ National Clinical Research Center for Neurological Diseases (China), Beijing, China; ^3^ Department of Neurosurgery, The Second Affiliated Hospital of Hainan Medical University, Haikou, China; ^4^ Beijing Neurosurgical Institute, Beijing Tiantan Hospital, Capital Medical University, Beijing, China; ^5^ Beijing Cancer Institute, Beijing Institute for Brain Disorders, Capital Medical University, Beijing, China; ^6^ Department of Neurosurgery, Xuanwu Hospital, Capital Medical University, Beijing, China; ^7^ Beijing Advanced Innovation Center for Big Data-Based Precision Medicine, Beihang University, Beijing, China

**Keywords:** newly diagnosed glioblastoma, extent of resection, non-contrast enhancing tumor, nomogram, prognosis

## Abstract

**Background:**

The extent of resection of non-contrast enhancing tumors (EOR-NCEs) has been shown to be associated with prognosis in patients with newly diagnosed glioblastoma (nGBM). This study aimed to develop and independently validate a nomogram integrated with EOR-NCE to assess individual prognosis.

**Methods:**

Data for this nomogram were based on 301 patients hospitalized for nGBM from October 2011 to April 2019 at the Beijing Tiantan Hospital, Capital Medical University. These patients were randomly divided into derivation (n=181) and validation (n=120) cohorts at a ratio of 6:4. To evaluate predictive accuracy, discriminative ability, and clinical net benefit, concordance index (C-index), receiver operating characteristic (ROC) curves, calibration curves, and decision curve analysis (DCA) were calculated for the extent of resection of contrast enhancing tumor (EOR-CE) and EOR-NCE nomograms. Comparison between these two models was performed as well.

**Results:**

The Cox proportional hazards model was used to establish nomograms for this study. Older age at diagnosis, Karnofsky performance status (KPS)<70, unmethylated O^6^-methylguanine-DNA methyltransferase (MGMT) status, wild-type isocitrate dehydrogenase enzyme (IDH), and lower EOR-CE and EOR-NCE were independent factors associated with shorter survival. The EOR-NCE nomogram had a higher C-index than the EOR-CE nomogram. Its calibration curve for the probability of survival exhibited good agreement between the identical and actual probabilities. The EOR-NCE nomogram showed superior net benefits and improved performance over the EOR-CE nomogram with respect to DCA and ROC for survival probability. These results were also confirmed in the validation cohort.

**Conclusions:**

An EOR-NCE nomogram assessing individualized survival probabilities (12-, 18-, and 24-month) for patients with nGBM could be useful to provide patients and their relatives with health care consultations on optimizing therapeutic approaches and prognosis.

## Introduction

Glioblastoma (GBM) is the most fatal and malignant of primary brain tumors in adults (1–3). Its highly aggressive behavior often proves clinically challenging to treat. Although advanced treatments have been applied in patients with newly diagnosed GBM (nGBM), satisfactory outcomes are rarely achieved. The high heterogeneity of GBM leads to a variable prognosis for these patients. Several clinical, imaging and biomarker studies have been employed to identify variable outcomes in patients with GBM. Karnofsky performance status (KPS), extent of surgical resection, O^6^-methylguanine-DNA methyltransferase (MGMT) methylation status and isocitrate dehydrogenase enzyme (IDH) mutation are the most uniformly documented prognostic factors in previous studies (4, 5).

Among these factors, the maximal safe surgical resection of contrast enhancing (CE) tumors has been recognized as a predominant treatment associated with prolonged survival in nGBM (6–8). Moreover, a number of studies on extending surgical resection to the non-contrast enhancing tumor (NCE) area further showed that reduced residual NCE tumor volume would be beneficial from survival (9, 10). Therefore, the extent of resection of NCE tumors (EOR-NCE) should not be neglected in our clinical practice.

Nomograms are an easy-to-use statistical model with representations of graphical diagrams that convey precise and individualized prognosis for multiple illnesses (11–14). This modality is also well-suited for highly heterogeneous GBM. The nomogram was first developed to identify new prognostic factors for survival in 2008 by Thierry Gorlia (15). Other established nomograms have since been widely used to predict prognosis in GBM (16, 17). Although these nomograms presented good prediction capacity, they did not include EOR-NCE for analysis.

To the best of our knowledge, we are the first to develop a nomogram including EOR-NCE in patients with nGBM. We hypothesized that the nomogram integrated with EOR-NCE would be more robust for identifying individual prognosis. The primary aim of this study was to assess the prognostic value of EOR-NCE for survival in patients with nGBM who received standard care of the Stupp protocol and to establish a nomogram integrated with the extent of surgical resection of NCE tumors for the prediction of these patients’ prognosis. Furthermore, a nomogram on the extent of resection of CE tumors (EOR-CE) was contrasted using the same dataset and compared to the EOR-NCE nomogram to gauge its predictive accuracy.

## Methods

### Data Collection and Study Population

A total of 301 patients in the Beijing Tiantan Hospital neurosurgical ward between October 2011 and April 2019 who were histologically diagnosed with nGBM and met inclusion criteria were included in our study. The eligibility criteria were (1) age at diagnosis≥18 years; (2) underwent neurosurgical resection with microscopic and histologic diagnosis as GBM; (3) received standard medical care primarily including maximum tumor resection, post-operative concurrent chemoradiotherapy and adjuvant chemotherapy according to the Stupp protocol (18); (4) had accessible clinical, imaging and molecular data for this analysis. Exclusion criteria: (1) multifocal or multicentric diseases; (2) participate in other clinical trials; (3) received secondary resection. All enrolled patients were randomly assigned into the training or testing cohort. All of them provided written informed consent, and this study was approved by the Ethical Committee of Beijing Tiantan Hospital.

### Tumor Volume Assessment on Magnetic Resonance Imaging

Pre-operative magnetic resonance imaging (MRI) scans were obtained within 1 week before tumor resection, and post-operative scans were performed within 48 to 72 h after surgery. Both pre-operative and post-operative CE and NCE tumor volumes were measured. T1-weighted post-contrast images were used for measuring CE tumor volume, and T2 or fluid-attenuated inversion-recovery (FLAIR) images were used to screen NCE tumor volume.

Regions of interest (ROIs) of each quantitative volume parameter for analysis were well drawn on contrast-enhancing T1-weighted sequences and T2 or FLAIR sequences (in cubic centimeter). All imaging parameters were independently assessed in consensus by two neuroradiologists (SJS and HCS) with more than 10 years’ experiences and were blinded to pathological and molecular diagnosis. And the average of 2 ROIs of each parameter was used to represent as its volume. Tumor volume was calculated by MRIcron software (www.nitrc.org). EOR was determined according to the following equation: (pre-operative tumor volume - post-operative tumor volume)/pre-operative tumor volume x 100%. T2 or FLAIR pre- and post-operative signals were carefully compared to corresponding slices’ diffusion weighted imaging (DWI) sequences when the ROI was drawn to distinguish surgery related edema or ischemia from residual tumor (10).

**Figure 1:**
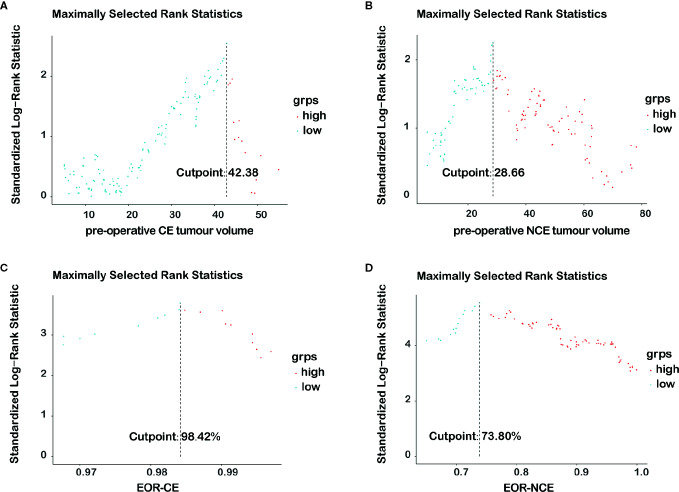
The distributions and optimal cutoff values of each imaging variable were shown in **(A–D)**.

**Figure 2:**
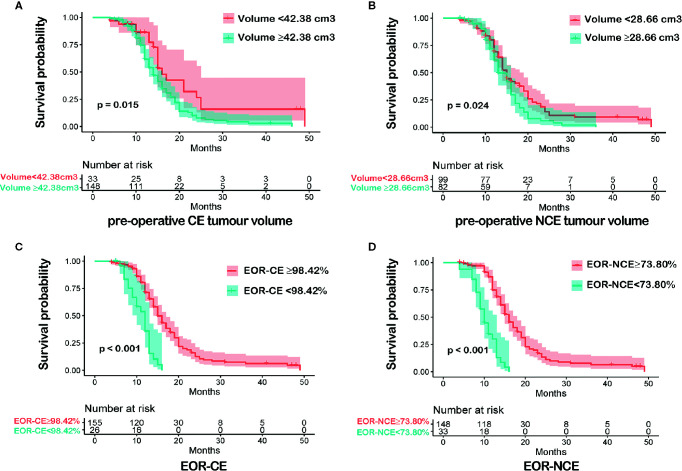
Kaplan-Meier survival analyses between each imaging variable subgroups according to the optimal cutoff value were shown in **(A–D)**. Patients with pre-operative CE tumour volume<42.38 cm^3^ had a longer overall survival (OS) than that of pre-operative CE tumour volume≥42.38 cm^3^, P = 0.015 **(A)**. Patients with pre-operative NCE tumour volume<28.66 cm^3^ exhibited a more favorable OS than that of pre-operative NCE tumour volume≥28.66 cm^3^, P = 0.024 **(B)**. Patients with EOR-CE≥98.42% showed a better OS than that of EOR-CE <98.42%, P < 0.001 **(C)**. Patients with EOR-NCE≥73.80% demonstrated a longer OS than that of EOR-NCE<73.80%, P < 0.001 **(D)**.

Our study chose the survminer package in R software to determine optimal cutoff values of imaging variables which were possible risk factors for outcome. The optimal cutoff values were calculated according to each continuous imaging variables, the survival time, and the survival status. These cutoff values were shown in **Figure 1** [pre-operative CE tumor volume (≥42.38 cm^3^ *vs <*42.38 cm^3^), pre-operative NCE tumor volume (≥28.66 cm^3^ *vs <*28.66 cm^3^) and EOR-CE (≥98.42% *vs <*98.42%) and EOR-NCE (≥73.80% *vs <*73.80%)]. The patients were then divided into high and low imaging variable subgroups according to the above optimal cutoff values. The overall survival analysis was performed between high and low imaging variable subgroups as well to confirm their prognostic difference (**Figure 2**). These cutoff values were used for both EOR-NCE and EOR-CE nomograms.

### Pathological Diagnosis and Molecular Detection

All pathological sections of these patients were re-evaluated by three neuropathologists (JMW, GLL and JD) according to the 2016 WHO classification of tumors of the CNS. MGMT promoter methylation and IDH mutation were detected by sequencing. The data were deposited in Dryad Digital Repository (doi:10.5061/dryad.xsj3tx9d4).

### Covariates Included

For the EOR-NCE nomogram, clinical variables that may be associated with survival of nGBM were recorded from the medical history by two neurosurgeons (PZ and XHC), including age (continuous), sex (male or female) KPS (<70 or ≥70), MGMT methylation (yes, no), IDH mutant (yes, no). Imaging variables included pre-operative CE tumor volume≥42.38 cm^3^ (yes, no), pre-operative NCE tumor volume≥28.66 cm^3^ (yes, no), EOR-CE≥98.42% (yes, no) and EOR-NCE≥73.80% (yes, no). Survival time was defined as the period from tumor resection to death or last follow-up. Survival status (alive or dead) at the 12-, 18- and 24-month time points was recorded according to patients’ follow-up results.

In addition, an EOR-CE nomogram was also established using the same cohorts of patients and statistical methods as for the EOR-NCE nomogram. The EOR-CE nomogram included all the above-mentioned prognostic factors for analysis, without EOR-NCE.

### Statistical Analyses

The statistical analysis was performed by R software (version 4.0.2, The R Foundation for Statistical Computing, Vienna, Austria). Continuous variables were compared by *t*-tests and categorical variables were assessed by chi-square tests. Survival analysis was calculated using the Kaplan–Meier method and log-rank test to compared the difference between derivation and validation cohorts. Univariate analysis was to evaluate the risk of possible factors, then these factors reached P<0.05 were subjected to Cox regression analysis. The nomogram was constructed according to the results of Cox regression analyses using the rms package. The final model was determined based on backward step-down selection process. The discrimination of the models was assessed in terms of the concordance index(C-index). Calibration curves were also drawn to evaluate the concordance between predicted and actual probabilities. Decision curve analyses (DCA) were employed to compare the benefits and improved performance of different models (19). Receiver operating characteristic (ROC) curves were conducted, and the area under the curve (AUC) values were calculated in order to compare the predictive efficacies of the different models. All tests were two-sided and were considered statistically significant when P<0.05.

## Results

### Patient Characteristics

**Figure 3:**
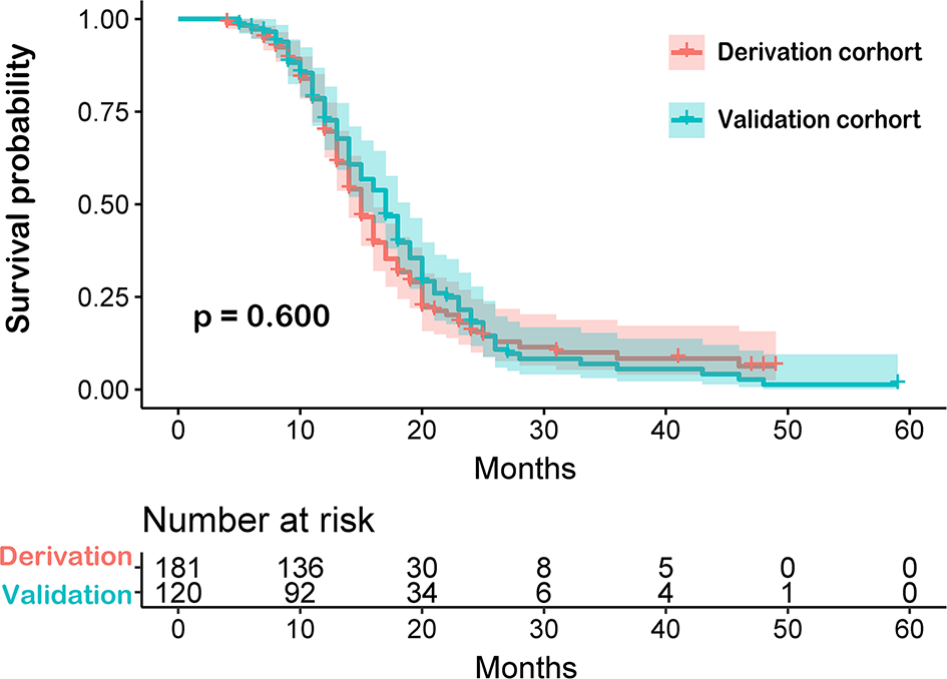
Comparison between derivation and validation cohorts of newly diagnosed glioblastoma patients by Kaplan-Meier survival curves. The overall survival time between these two cohorts was not significant.

In this study, we enrolled 301 patients from Beijing Tiantan Hospital. Patients were randomly sub-grouped into either the training cohort (n=181) or the testing cohort (n=120) in a 6 to 4 ratio, respectively. Both demographics and clinical characteristics of each group were compared as shown in **Table 1**. No statistically significant differences in these characteristics were observed between the derivation and validation cohorts. The overall survival time between these two cohorts was not significant (P=0.600, **Figure 3**).

**Table 1:** Baseline characteristics of enrolled patients with newly diagnosed glioblastoma in the derivation and external validation cohorts.

Characteristic	Derivation set (n=181)	Validation set (n=120)	P-value
Age at diagnosis (mean (SD) [Range])	53.31 ± 12.63 (18-80)	52.00 ± 13.69 (18-75)	0.393
Sex (N %)			
Male	123 (68.0)	74 (61.7)	0.261
Female	58 (32.0)	46 (38.3)	
KPS (N %)			
≥70	154 (84.1)	102 (85.0)	0.984
<70	27 (14.9)	18 (15.0)	
Tumor location (N %)			
Frontal	62 (34.3)	48 (39.7)	0.788
Temporal	60 (33.1)	33 (27.3)	
Parietal	34 (18.8)	24 (19.8)	
Occipital	15 (8.3)	8 (6.6)	
Others	10 (5.5)	7 (5.8)	
IDH status (N %)			
Mutant	20 (11.0)	12 (10.0)	0.772
Wild-type	161 (89.0)	108 (90.0)	
MGMT methylation (N %)			
Unmethylated	95 (52.5)	56 (46.7)	0.323
Methylated	86 (47.5)	64 (53.3)	
Pre-operative volume, cm^3^			
CE tumors			
Mean (SD)	28.29 ± 22.75	31.02 ± 20.04	0.287
Range	0.67-122.40	1.16-84.13	
NCE tumors			
Mean (SD)	40.15 ± 33.56	40.67 ± 30.20	0.892
Range	0.30-201.11	1.53-147.49	
Post-operative volume, cm^3^			
CE tumors			
Mean (SD)	0.30 ± 1.01	0.38 ± 0.93	0.468
Range	0.00-8.12	0.00-4.50	
NCE tumors			
Mean (SD)	5.12 ± 9.05	5.11 ± 11.58	0.993
Range	0.00-54.90	0.00-100.73	
Extent of resection (%)			
CE tumors			
Mean (SD)	98.57 ± 5.15	98.93 ± 27.99	0.556
Range	60.74-100.00	81.43-100.00	
NCE tumors			
Mean (SD)	88.48 ± 16.66	88.81 ± 16.43	0.906
Range	0.00-100.00	3.02-100.00	

CE, contrast enhanced; NCE, non–contrast enhanced; IDH, isocitrate dehydrogenase gene; KPS, Karnofsky Performance Score; MGMT, O6-methylguanine-DNA methyltransferase.

### Univariate Analyses for the Derivation Cohort of Patients With nGBM

Age at diagnosis, sex, KPS, IDH mutation status, MGMT methylation status, pre-operative CE tumor volume, pre-operative NCE tumor volume, EOR-CE and EOR-NCE were possible prognostic risk factors for univariate analysis for the derivation cohort of patients with nGBM. Results showed that older age, KPS<70, unmethylated MGMT, wild type IDH, larger pre-operative CE and NCE tumor volume, lower EOR-CE and EOR-NCE were significantly associated with increased risk of mortality in the derivation cohort (all P<0.05), but sex did not exhibit a significant difference (P=0.178). Both EOR-NCE and EOR-CE nomograms used the same cohort of patients and cutoff values of variables for univariate analysis. The included variables for each nomogram were presented in **Supplemental Table 1**. Then variables with significant difference were next subjected to multivariate Cox regression analyses for the two models, respectively.

### Multivariate Analyses of EOR-NCE and EOR-CE Nomograms

For the EOR-NCE nomogram, multivariate Cox regression analyses indicated that older age (HR=1.019, 95% confidence interval [CI]: 1.003–1.035, P=0.020), KPS≥70 (HR=0.455, 95% CI: 0.280–0.739, P=0.001), methylated MGMT (HR=0.539, 95% CI: 0.372–0.780, P=0.001), IDH mutant (HR=0.311, 95% CI: 0.160–0.606, P <0.001), EOR-CE≥98.42% (HR=0.457, 95% CI: 0.249–0.837, P=0.011) and EOR-NCE≥73.80% (HR=0.149, 95% CI: 0.256–0.873, P<0.001) were independent prognostic factors for outcomes (**Table 2**).

**Table 2:** Multivariate Cox regression analyses in patients with newly diagnosed GBM for the derivation cohort of EOR-NCE and EOR-CE nomogram.

Characteristic	EOR-NCE		EOR-CE	
	HR (95% CI)	P-value	HR (95% CI)	P-value
Age (yr)	1.019 (1.003–1.035)	0.020	1.016 (1.000–1.032)	0.047
KPS≥70	0.455 (0.280–0.739)	0.001	0.476 (0.294–0.769)	0.002
IDH mutant	0.311 (0.160–0.606)	P<0.001	0.301 (0.155–0.582)	P<0.001
MGMT methylated	0.539 (0.372–0.780)	0.001	0.637 (0.445–0.912)	0.013
Extent of resection (%)				
CE≥98.42	0.457 (0.249–0.837)	0.011	0.281 (0.157–0.501)	P<0.001
NCE≥73.80	0.149 (0.256–0.873)	P<0.001	NA	NA

CE, contrast enhanced; NCE, non–contrast enhanced; IDH, isocitrate dehydrogenase gene; KPS, Karnofsky Performance Score; MGMT, O6-methylguanine-DNA methyltransferase; NA, not available.

For the EOR-CE nomogram, multivariate Cox regression analyses showed that older age (HR=1.016, 95% CI: 1.000–1.032, P=0.047), KPS≥70 (HR=0.476, 95% CI: 0.294–0.769, P=0.002), methylated MGMT (HR=0.637, 95% CI: 0.445–0.912, P=0.013), IDH mutant (HR=0.301, 95% CI: 0.155–0.582, P<0.001), EOR-CE≥98.42% (HR=0.281, 95% CI: 0.157–0.501, P<0.001) were independent prognostic factors for survival (**Table 2**).

### Derivation and Validation of EOR-NCE and EOR-CE Prognostic Nomograms

**Figure 4:**
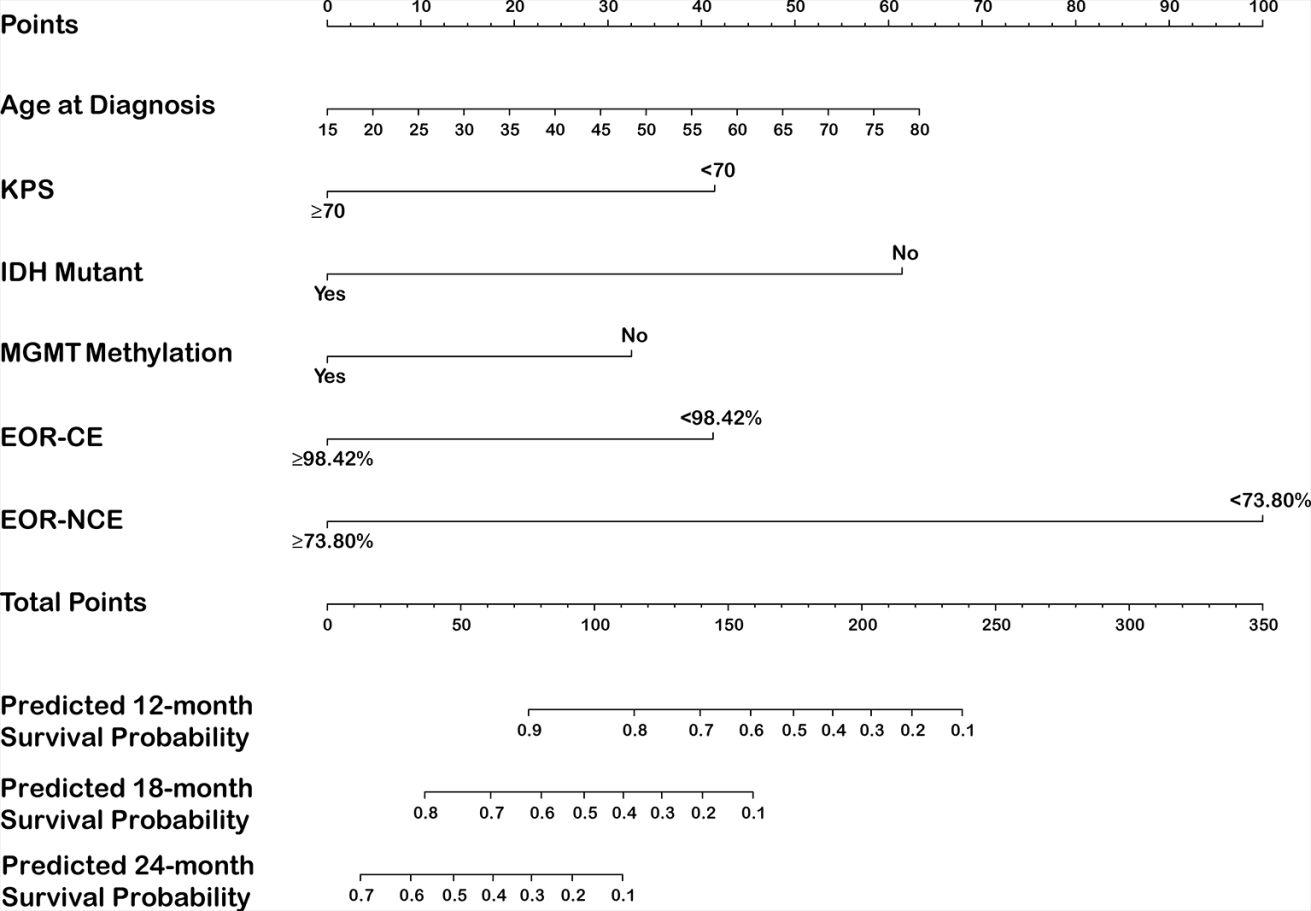
Nomogram for predicting 12-, 18-, and 24-month survival in newly diagnosed glioblastoma patients integrated with EOR-NCE.

The EOR-NCE and EOR-CE prognostic nomogram were constructed according to coefficients of the Cox multivariate regression analysis (**Figure 4** and **Supplemental Figure 1**, respectively). Each factor’s single score presenting on a point scale axis was summed for total score. Then, the total score was used to determine the likelihood of 12-, 18-, and 24-month survival probabilities for individual patients based on the total point scale axis.

**Figure 5:**
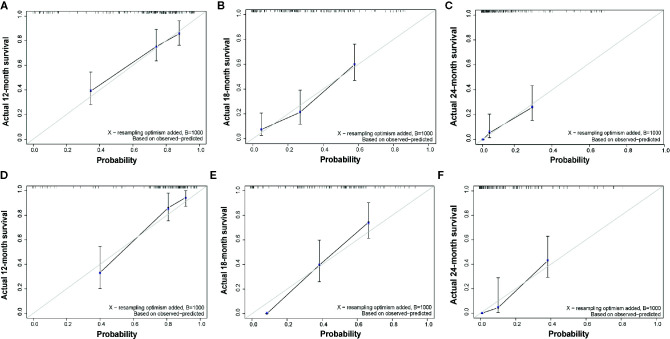
Calibration curve of overall survival at 12-, 18-, and 24-months for the derivation **(A–C)** and validation **(D–F)** cohorts for EOR-NCE nomogram, showing that there was high consistency between the nomogram’s predicted probability and actual survival probability. The nomogram-predicted probability of survival and actual survival are plotted on the x-axis and y-axis, respectively.

The C-index of the EOR-NCE nomogram was 0.779 (95% CI, 0.733–0.824, P<0.05) in the derivation cohort. Moreover, to verify the validity of the derivation nomogram, we developed a random cohort of patients with nGBM. The C-index of the validation nomogram was 0.790 (95% CI, 0.743–0.838, P<0.05). Additionally, the calibration curve showed that there was high consistency between the nomogram’s predicted probability and actual survival probability for 12-, 18-, and 24-month survival (**Figure 5**).

Similarly, we also calculate the predictive parameters of EOR-CE nomogram. The C-index was 0.727 (95% CI, 0.681–0.774, P<0.05)] in derivation cohort. And the C-index was 0.748 (95% CI, 0.699–0.797, P<0.05) in validation cohort. Besides, the calibration curve showed a modest consistency between the nomogram’s predicted probability and actual survival probability for 12-, 18-, and 24-month survivals (**Supplemental Figure 2**).

### Comparison of the Performance Between EOR-CE and EOR-NCE Nomograms

**Figure 6:**
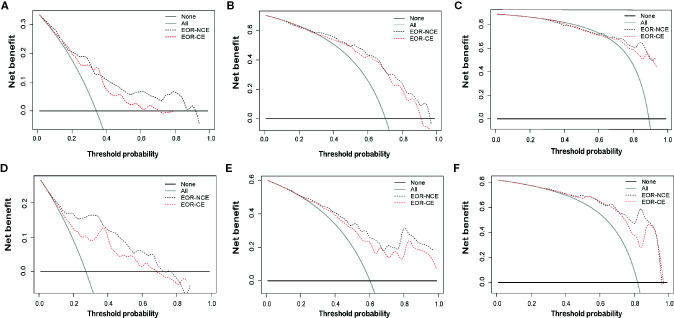
Decision curve analysis (DCA) at 12-, 18-, and 24-months for the derivation **(A–C)** and validation **(D–F)** cohorts. DCAs also showed the difference of net benefit between EOR-NCE and EOR-CE nomograms. The horizontal black line represents no patients experiencing a survival event, while the grey line represents all patients reaching the survival endpoint. The decision curve analysis of the EOR-NCE nomogram provides better net benefit than the EOR-CE models across a range of threshold probabilities.

The EOR-NCE nomogram showed superior predictive ability compared to that of the EOR-CE nomogram for nGBM, supported by the evaluative parameters. First, C-indexes of these two models with statistical significance were compared to assess their concordance. We found the C-index of the EOR-NCE nomogram was higher than for the EOR-CE in both the derivation (0.779 *vs* 0.727) and validation (0.790 *vs* 0.748) cohorts, illustrating that this nomogram was well suited for predicting survival in nGBM patients. Second, on decision curve analysis (DCA), the EOR-NCE nomogram provided superior net benefit and improved performance for the 12-, 18-, and 24-month prognostic assessments compared to EOR-CE models in the derivation (**Figures 6A–C**) and validation (**Figures 6D–F**) cohorts. Last but not least, in the established ROC, AUC of the EOR-NCE nomogram was higher than that in the EOR-CE nomogram (0.730 *vs* 0.656 for 12-month, 0.803 *vs* 0.756 for 18-month, 0.803 *vs* 0.763 for 24-month in derivation cohort; 0.870 *vs* 0.809 for 12-month, 0.848 *vs* 0.821 for 18-month, 0.920 *vs* 0.902 for 24-month in validation cohort, **Table 3**).

**Table 3:** The predictive discrimination ability of the EOR-NCE nomogram compared to EOR-CE nomogram in the derivation and validation cohorts.

	C-index (95% CI)	Area Under the Curve (AUC)		
		12 months	18 months	24 months
Derivation cohort (n=181)				
EOR-NCE	0.779 (0.733-0.824)	0.730	0.803	0.803
EOR-CE	0.727 (0.681-0.774)	0.656	0.756	0.763
Validation cohort (n=120)				
EOR-NCE	0.790 (0.743-0.838)	0.870	0.848	0.920
EOR-CE	0.748 (0.699-0.797)	0.809	0.821	0.902

EOR-NCE, non-contrast enhancing tumor; EOR-CE, contrast enhancing tumor; AUC, area under the curve.

## Discussion

Patients with nGBM inevitably suffer a very poor prognosis. Only approximately 5% of patients survive beyond 5 years (1, 3). Therefore, precise evaluation of individual prognosis is of high value for treatment decision-making and providing patients with consultation on intervals of follow-up and prognosis. Given that nomograms are commonly used for prediction of survival among many kinds of cancer in a visual manner (11–14), we created nomograms to estimate individual survival probability at 12-, 18-, 24-months in nGBM and included clinical, imaging, molecular factors and EOR of CE and NCE tumors as prognostic variables. Our nomogram model was simple and clinically practical since all risk factors were easily accessible for clinical use to predict survival.

In previous nomograms on nGBM, a series of prognostic factors associated with survival were documented. Gorlia et al. constructed an easy-to-use nomogram for nGBM patients showing that MGMT promoter methylation status was significant for prognosis, as well as age at diagnosis, performance status and EOR (15). Similar results were also reported by Molitoris et al (20). In addition, Haley et al. used gender for analysis and found that age at diagnosis, KPS, EOR and MGMT promoter methylation status were all prognostic factors (16). Then, they added concurrent chemoradiotherapy and confirmed its predictive value in a subsequent nomogram of a wild type IDH nGBM cohort (17). Although there remain some nomograms that were developed using other prognostic factors (20–22), little attention has been paid to the extent of neurosurgical resection of NCE.

To the best of our knowledge, we are the first to construct an individual nomogram including the prognostic variable of EOR-NCE for nGBM. There were several reasons we made this choice. First, GBM cells infiltrate beyond the CE component and form NCE lesions containing higher tumor burden than that of the CE area (23). Moreover, with the increased understanding of the NCE component, many neurosurgical studies have recommended the extended strategy of removing the NCE component of GBM rather than focusing only on the EOR-CE (9, 24, 25). Subsequently, several studies further confirmed that patients with GBM who underwent aggressive resection of NCE tumors achieved longer survival than patients who only received gross total resection of the CE component (10, 26, 27). Therefore, supported by these findings, we evaluated the EOR-NCE in the current nomogram.

Consistently, our proposed model suggested that older age at diagnosis, KPS<70, unmethylated MGMT, IDH wild-type, lower EOR-CE and EOR-NCE were independently associated with decreased survival (10, 15–17, 20, 26, 27). However, our study did not identify significant prognostic significance for male gender (15, 16, 20), pre-operative CE tumor volume or pre-operative NCE tumor volume (28–32), which were controversial in previous studies.

In this study, the EOR-NCE nomogram was well suited for estimating survival probability, as supported by the C-index (0.779 for divarication and 0.790 for validation cohorts) and the good agreement calibration curves. Furthermore, we compared the EOR-NCE nomogram to the EOR-CE nomogram to confirm their predictive value. As expected, in both derivation and validation cohorts the EOR-NCE nomogram showed superior predictive abilities compared to the EOR-CE model for survival probability in C-index and the AUC; moreover, the EOR-NCE nomogram exhibited better net benefit and improved performance for 12-, 18-, and 24-month prognostic assessment compared to EOR-CE nomogram. The AUC of the EOR-NCE nomogram was higher than in the EOR-CE nomogram 12-, 18-, and 24-months. This result also demonstrated that the EOR-NCE nomogram much was better than the EOR-CE nomogram for survival prediction.

There are several limitations in our study. Firstly, although patients in this nomogram were randomly assigned to the derivation and validation cohorts, they were from a single center without including their races for analysis. To make the predictive model widely applicable, it needs additional datasets from other centers to validate the predictive efficiency of our derivation model. Secondly, in order to reduce bias manual segmentation in clinical use, CE and NCE tumor volumes should be automatically measured by advanced computer technologies in the future. Besides, IDH mutation in our study is only relative to IDH1 gene alterations, since IDH2 gene mutants are rare in GBM (33). The predictive value of IDH2 remains to be explored in future nomograms. IDH1 mutation is 10% to 11% in our cohort was mildly higher than the previous report (10%) (34), which may have been caused by selection bias of these enrolled patients. Moreover, the sample size of our study was small; therefore, we will enlarge the numbers in our cohorts to further confirm the accuracy of the current nomogram model. This study included patients with nGBM who received standard treatment for this derivation and validation model. One reason is that concurrent chemoradiotherapy with temozolomide has been advanced all over the world as standard of care since 2005 and prolongs survival (18). Additionally, the uniform post-operative treatment allowed full evaluation of the prognostic value of the EOR-NCE nomogram. Therefore, our study did not include patients with nGBM who underwent monotherapy of either chemotherapy or radiotherapy.

## Conclusions

This nomogram integrated with EOR-NCE to examine survival probability in nGBM patients was developed and independently validated. In addition, the EOR-NCE nomogram exhibited better performance than the EOR-CE nomogram in predicting survival. Our nomogram has important clinical and prognostic significance in assessment of individualized survival rather than of group evaluation. We believe this model will have important use for providing health consultations for patients and their relatives with respect to treatment decisions and prognosis.

Free online tool for implementing EOR-NCE nomogram is provided: https://ttns-6.shinyapps.io/nomogram_for_gbm_patients/.

## Data Availability Statement

The data were deposited in Dryad Digital Repository (doi: 10.5061/dryad.xsj3tx9d4).

## Ethics statement

The studies involving human participants were reviewed and approved by This study was approved by the Ethical Committee of Beijing Tiantan Hospital, Capital Medical University. The patients/participants provided their written informed consent to participate in this study.

## Author Contributions

ZZ: design study, analysis and manuscript drafting. ZJ: data analysis and plotting of figures. DL, YZ, CL, YM, XC, and JF: clinical data collection and follow-up of patients. YW and SH: manuscript preparation, revising and language polish. NJ: study design, approved the ﬁnal version of the manuscript and study supervision. All authors contributed to the article and approved the submitted version.

## Funding

This work was supported by Capital Characteristic Clinical Application Project (Z181100001718196).

## Conflict of Interest

The authors declare that the research was conducted in the absence of any commercial or financial relationships that could be construed as a potential conflict of interest.

## Supplementary Material

Supplementary Figure 1 Nomogram for predicting 12-, 18-, and 24-month survival in newly diagnosed glioblastoma patients integrated with EOR-CE.

Supplementary Figure 2 Calibration curve of overall survival at 12-, 18-, and 24-months for the derivation (A-C) and validation (D-F) cohorts for EOR-CE nomogram, showing a modest consistency between the nomogram’s predicted probability and actual survival probability. The nomogram-predicted probability of survival and actual survival are plotted on the x-axis and y-axis, respectively.
