# Supplementary figures and images for: A Nomogram Predicts Individual Prognosis in Patients With Newly Diagnosed Glioblastoma by Integrating the Extent of Resection of Non-Enhancing Tumors
Source: Front Oncol. 2020 Dec 2;10:598965. doi: 10.3389/fonc.2020.598965 (PMC7739947; doi:10.3389/fonc.2020.598965)

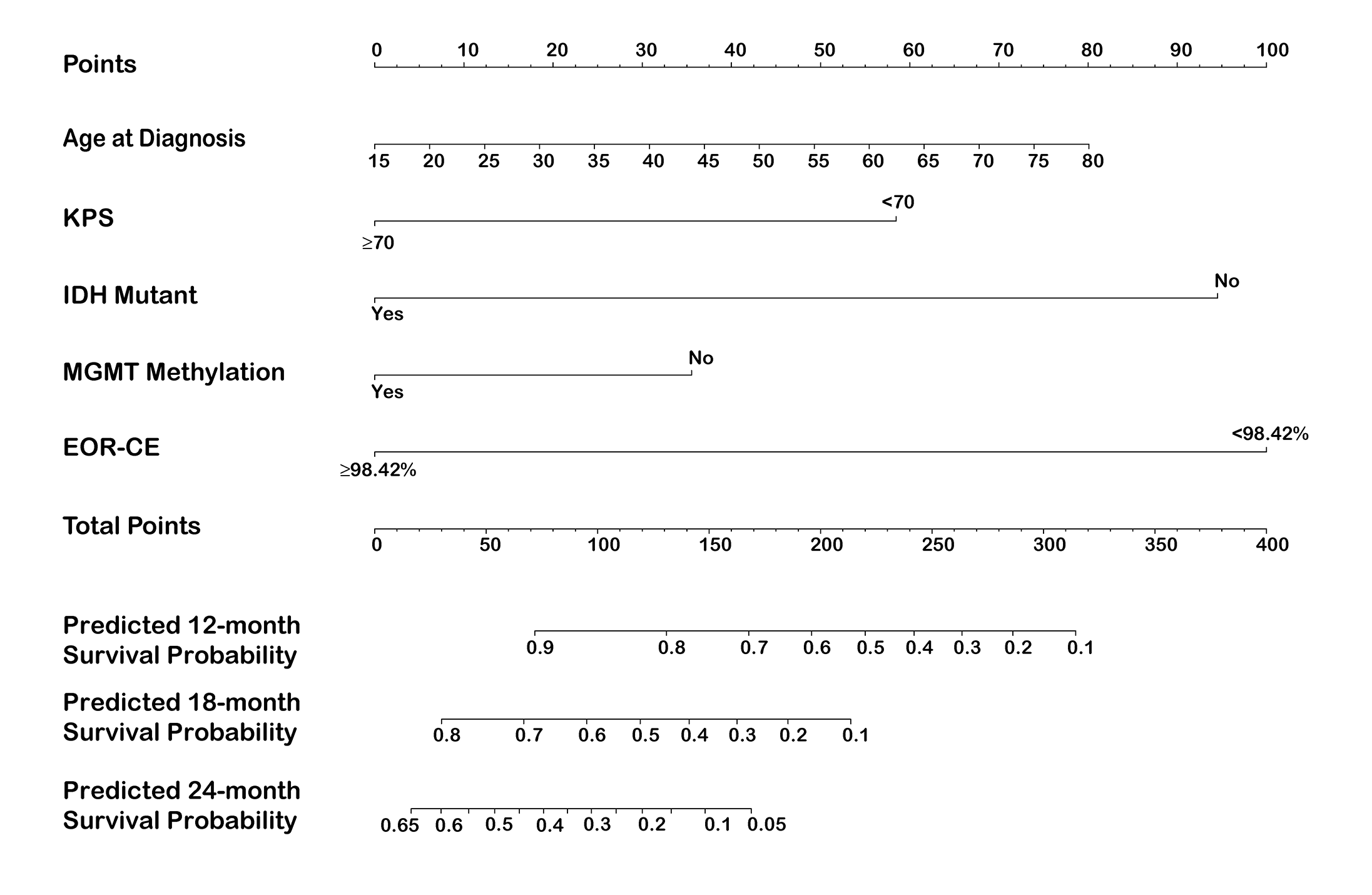

Supplement: Supplementary Figure 1 — Nomogram for predicting 12-, 18-, and 24-month survival in newly diagnosed glioblastoma patients integrated with EOR-CE. [file Image_1.tif]

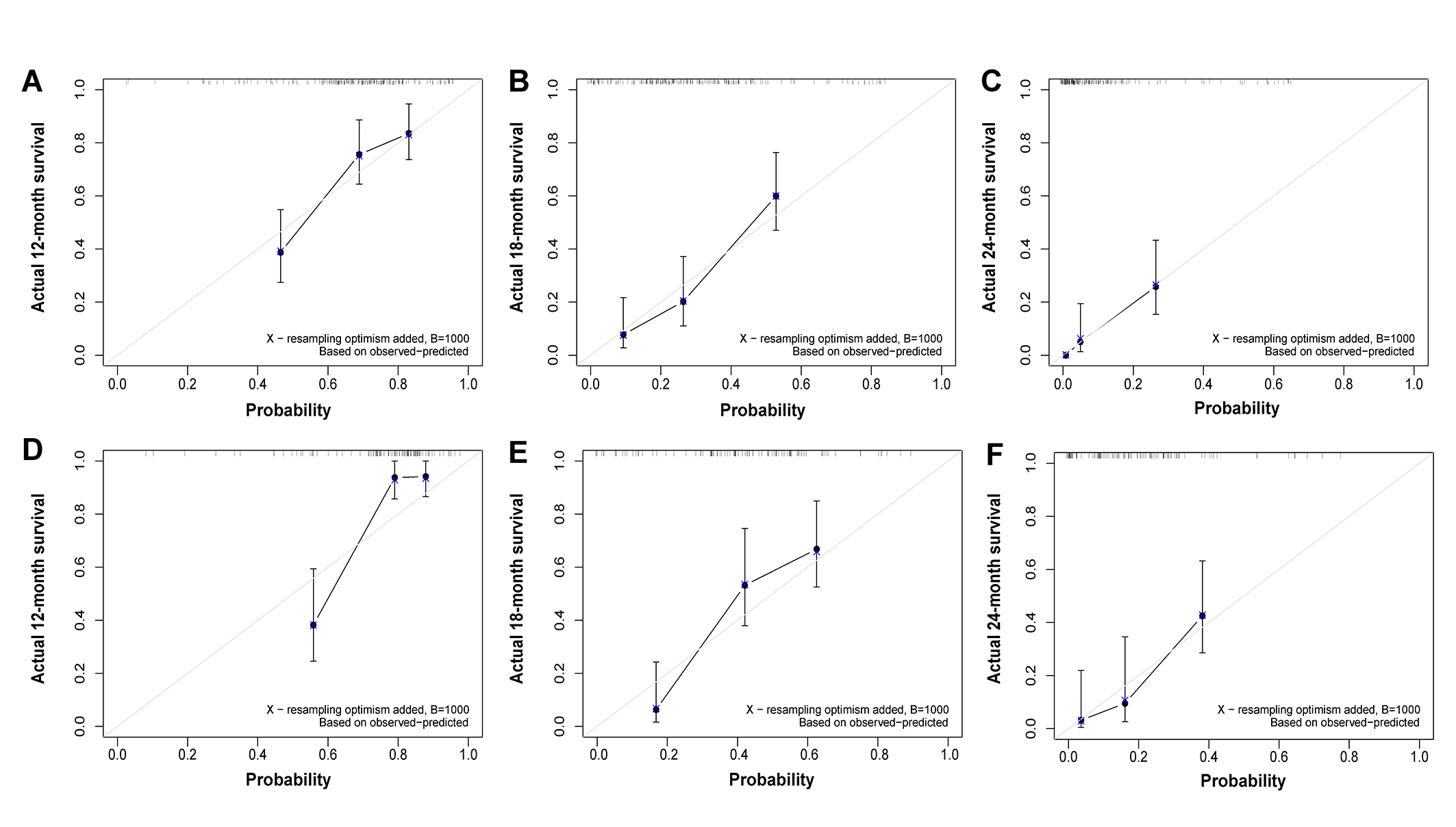

Supplement: Supplementary Figure 2 — Calibration curve of overall survival at 12-, 18-, and 24-months for the derivation (A-C) and validation (D-F) cohorts for EOR-CE nomogram, showing a modest consistency between the nomogram’s predicted probability and actual survival probability. The nomogram-predicted probability of survival and actual survival are plotted on the x-axis and y-axis, respectively. [file Image_2.tif]
