# Supplementary material for: A Nomogram Predicts Individual Prognosis in Patients With Newly Diagnosed Glioblastoma by Integrating the Extent of Resection of Non-Enhancing Tumors
Source: Front Oncol. 2020 Dec 2;10:598965. doi: 10.3389/fonc.2020.598965 (PMC7739947; doi:10.3389/fonc.2020.598965)
Supplement: Supplementary file 3 [file Table_1.docx]

**Supplemental Table 1 Univariate analyses for the derivation cohort of patients with nGBM**

|  | Univariate analysis for both EOR-NCE and EOR-CE nomograms | |
| --- | --- | --- |
| Characteristic | HR (95% CI) | P-value |
| Age (yr) | 1.027(1.012-1.041) | P<0.001 |
| Male sex | 1.287(0.892-1.858) | 0.178 |
| KPS≥70 | 0.460(0.291-0.726) | P<0.001 |
| Tumor location |  |  |
| Frontal | reference |  |
| Temporal | 1.182(0.769-1.818) | 0.446 |
| Parietal | 1.101(0.658-1.841) | 0.714 |
| Occipital | 1.115(0.605-2.056) | 0.728 |
| Others | 2.198(0.969-4.985) | 0.059 |
| IDH mutant | 0.263(0.139-0.496) | P<0.001 |
| MGMT methylated | 0.637(0.426-0.954) | 0.029 |
| Pre-operative volume, cm^3^ |  |  |
| CE tumours≥42.38 | 1.871(1.128-3.105) | 0.015 |
| NCE tumours≥28.66 | 1.506(1.055-2.150) | 0.024 |
| Extent of resection (%) |  |  |
| CE≥98.42 | 0.281(0.158-0.499) | P<0.001 |
| NCE≥73.80 | 0.151(0.093-0.249) | P<0.001* |

**Abbreviations:** CE, contrast enhanced; NCE, non-contrast enhanced; IDH, isocitrate dehydrogenase gene; KPS, Karnofsky Performance Score; MGMT, O^6^-methylguanine-DNA methyltransferase. * Univariate analysis of EOR-NCE was only for construction of EOR-NCE nomogram, while it was not employed for establishing EOR-CE nomogram.
